# Supplementary figures and images for: ATP Is a Major Determinant of Phototrophic Bacterial Longevity in Growth Arrest
Source: mBio. 2023 Feb 14;14(2):e03609-22. doi: 10.1128/mbio.03609-22 (PMC10128053; doi:10.1128/mbio.03609-22)

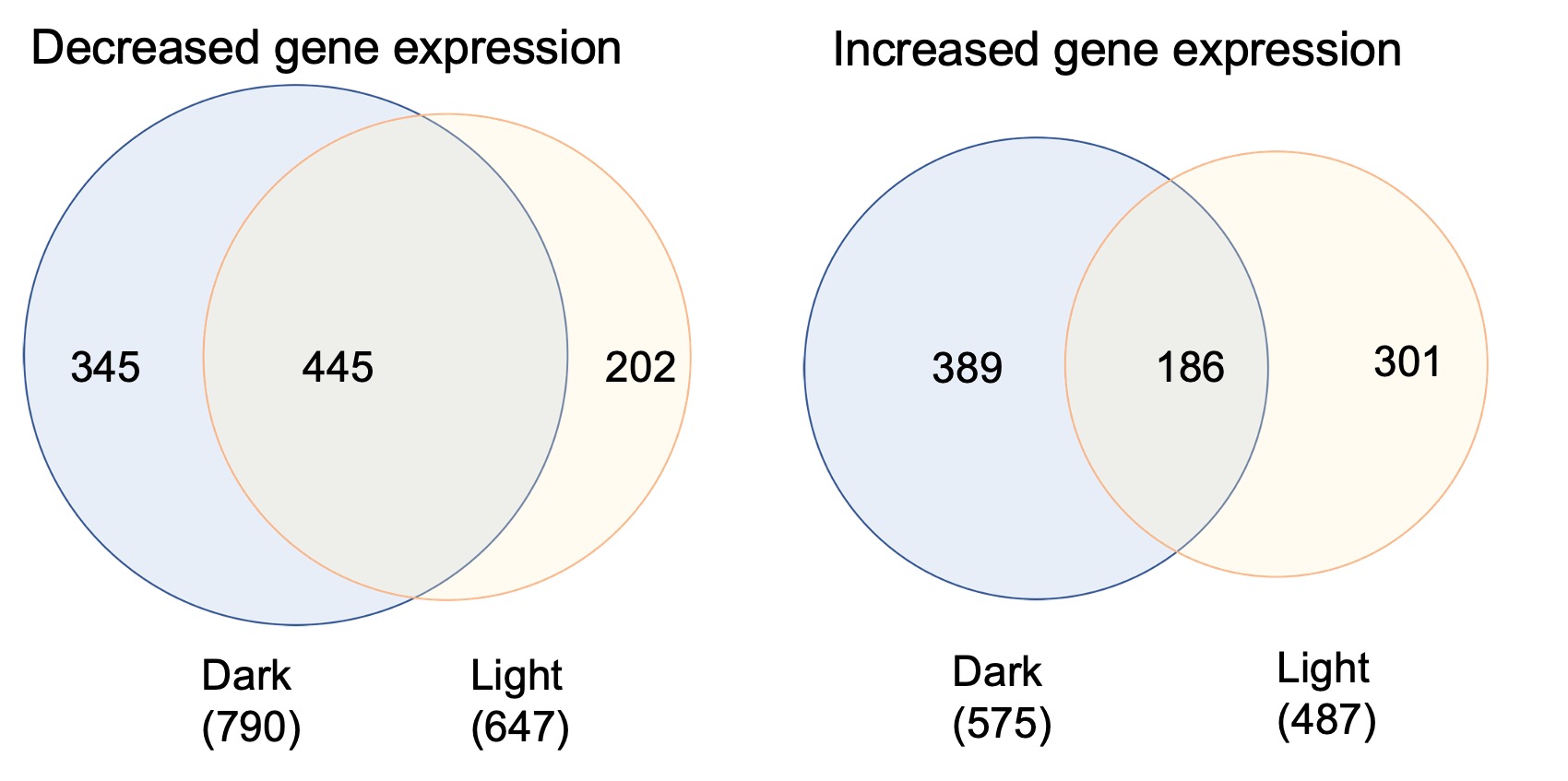

Supplement: FIG S1 [file mbio.03609-22-s0001.jpg]

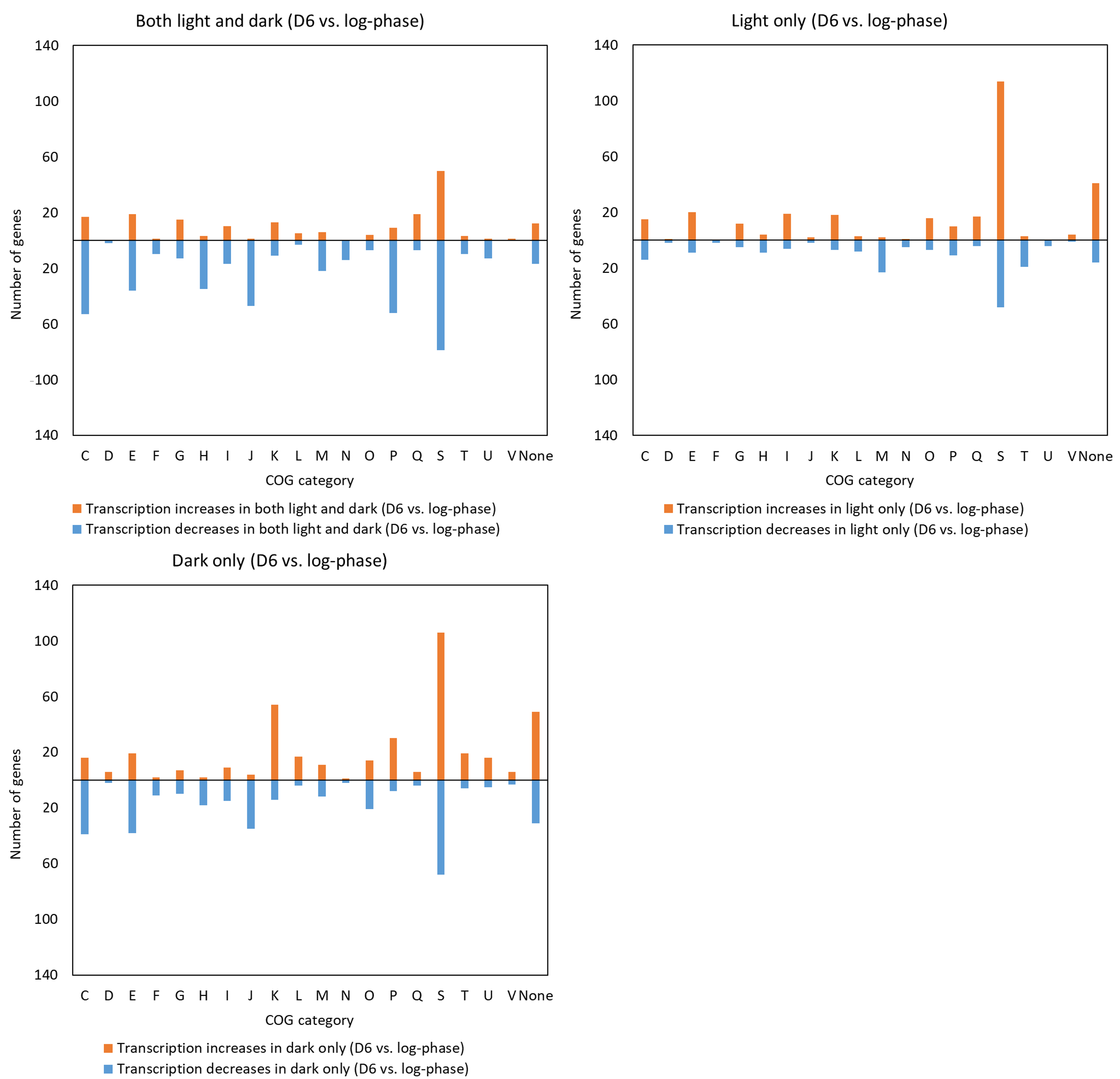

Supplement: FIG S2 [file mbio.03609-22-s0002.tif]
