# Supplementary material for: ATP Is a Major Determinant of Phototrophic Bacterial Longevity in Growth Arrest
Source: mBio. 2023 Feb 14;14(2):e03609-22. doi: 10.1128/mbio.03609-22 (PMC10128053; doi:10.1128/mbio.03609-22)
Supplement: TABLE S1 [file mbio.03609-22-s0003.pdf]

SI Table S1. Number of reads generated by RNA sequencing before and after quality-filtering and alignment to the reference transcriptome. Average numbers of paired-end reads are displayed along with standard deviations of two biological replicates (SD).

**Timepoint**	**Incubation Condition**	**Average raw paired-end reads (± SD)**	**Average quality-filtered paired-end reads aligned to transcriptome (± SD)**
Log-phase growth	Continuous light	26,136,929 (± 508,699)	15,318,451 (± 853,900)
Growth arrest day 1	Continuous light	27,004,942 (± 4,022,217)	14,705,789 (± 2,485,698)
Growth arrest day 6	Continuous light	20,268,622 (± 3,355,763)	10,887,197 (± 1,603,061)
Growth arrest day 6	Continuous darkness	15,615,249 (± 582,303)	7,034,937 (± 646,588)
Growth arrest day 20	Continuous light	22,298,704 (± 1,400,215)	12,536,491 (± 639,008)
